# Multidisciplinary Predialysis Education Reduced the Inpatient and Total Medical Costs of the First 6 Months of Dialysis in Incident Hemodialysis Patients

**DOI:** 10.1371/journal.pone.0112820

**Published:** 2014-11-14

**Authors:** Yu-Jen Yu, I-Wen Wu, Chun-Yu Huang, Kuang-Hung Hsu, Chin-Chan Lee, Chio-Yin Sun, Heng-Jung Hsu, Mai-Szu Wu

**Affiliations:** 1 Department of Nephrology, Chang Gung Memorial Hospital, Keelung, Taiwan; 2 College of Medicine, Chang Gung University, Tao-Yuan, Taiwan; 3 Laboratory for Epidemiology, Department of Health Care Management, Chang Gung University, Tao-Yuan, Taiwan; 4 Division of Nephrology, Taipei Medical University Hospital, Taipei, Taiwan; 5 School of Medicine, Taipei Medical University, Taipei, Taiwan; University of Sao Paulo Medical School, Brazil

## Abstract

**Background:**

The multidisciplinary pre-dialysis education (MPE) retards renal progression, reduce incidence of dialysis and mortality of CKD patients. However, the financial benefit of this intervention on patients starting hemodialysis has not yet been evaluated in prospective and randomized trial.

**Methods:**

We studied the medical expenditure and utilization incurred in the first 6 months of dialysis initiation in 425 incident hemodialysis patients who were randomized into MPE and non-MPE groups before reaching end-stage renal disease. The content of the MPE was standardized in accordance with the National Kidney Foundation Dialysis Outcomes Quality Initiative guidelines.

**Results:**

The mean age of study patients was 63.8±13.2 years, and 221 (49.7%) of them were men. The mean serum creatinine level and estimated glomerular filtration rate was 6.1±4.0 mg/dL and 7.6±2.9 mL⋅min^−1^⋅1.73 m^−2^, respectively, at dialysis initiation. MPE patients tended to have lower total medical cost in the first 6 months after hemodialysis initiation (9147.6±0.1 USD/patient vs. 11190.6±0.1 USD/patient, p = 0.003), fewer in numbers [0 (1) vs. 1 (2), p<0.001] and length of hospitalization [0 (15) vs. 8 (27) days, p<0.001], and also lower inpatient cost [0 (2617.4) vs. 1559,4 (5019.6) USD/patient, p<0.001] than non-MPE patients, principally owing to reduced cardiovascular hospitalization and vascular access–related surgeries. The decreased inpatient and total medical cost associated with MPE were independent of patients' demographic characteristics, concomitant disease, baseline biochemistry and use of double-lumen catheter at initiation of hemodialysis.

**Conclusions:**

Participation of multidisciplinary education in pre-dialysis period was independently associated with reduction in the inpatient and total medical expenditures of the first 6 months post-dialysis owing to decreased inpatient service utilization secondary to cardiovascular causes and vascular access–related surgeries.

**Trial Registration:**

ClinicalTrials.gov NCT00644046

## Introduction

The number of patients worldwide with chronic kidney disease (CKD) and end-stage renal disease (ESRD) being treated with renal replacement therapy has been continuously increasing in recent years, with a 7% rate of increase per year [1]. Aging and type 2 diabetes mellitus are the two most important factors [2]. Taiwan is the leading country in terms of ESRD prevalence, with a rate of 2447 per million population [3]. The implementation of National Health Insurance (NHI) has helped drive the growth of the ESRD populations in Taiwan [4]. However, the official prohibition of the use of aristolochic acid–containing herbs and the introduction of the nationwide CKD Preventive Project with a multidisciplinary care program have proved their effectiveness in decreasing the incidence of dialysis, and mortality and medical costs of CKD patients [5]. However, the financial benefit of this intervention has not yet been evaluated in prospective and randomized manner on patients starting hemodialysis.

High ESRD prevalence constitutes a large economic burden for the patient, society, and the country. Renal insufficiency represents a status of increased cardiovascular disease risk, comorbidities, and mortality [6], demanding high medical expenditures and health-care utilization [1]. The cost for ESRD has increased to $34.3 billion, accounting for 6.3% of the total Medicare budget according to the 2013 US Renal Data System Annual Data Report [7]. Similarly, in Taiwan, the annual dialysis costs have accounted for 5.0–7.52% of the total budget of the NHI in recent years [8]. Optimal and efficient treatment strategies to combat the high prevalence of ESRD and its high cost of care are thus urgently needed.

Predialysis education can decrease the ESRD incidence and mortality in the first year of dialysis [9], [10]. Nephrology-based care has also significantly improved the clinical outcomes of CKD patients in both the predialysis and postdialysis periods [11], [12], especially in type 2 diabetes patients [13], [14]. It has been associated with better biochemical variables, shorter hospitalization length, a higher percentage of elective construction of the arteriovenous fistula, and the availability of alternative dialysis modality [15]. Our previous controlled cohort study has confirmed that multidisciplinary predialysis education (MPE) based on the National Kidney Foundation Dialysis Outcomes Quality Initiative (NKF/DOQI) guidelines provides a better outcome with a significantly reduced incidence of ESRD and all-cause mortality [16]. The MPE program has effectively improved the quality of pre-ESRD care, increased patients' self-care ability, and has retarded renal progression and reduced morbidities in late-stage CKD patients [17]. Multidisciplinary predialysis team care was found to decreased service utilization and saved medical costs in the 6 months before dialysis initiation and at dialysis initiation, being secondary to the early preparation of vascular access and the lack of hospitalization at dialysis initiation [18]. Predialysis nephrology–based care has been associated with reduced costs in elderly patients after the initiation of dialysis [19]. Most of these controlled or randomized trials have used the renal or patient outcome as their endpoints. However, randomized studies to evaluate the cost-saving effect of MPE in the post-dialysis period have been seldom reported to date.

Most of the medical costs associated with caring for CKD patients are incurred for the treatment of comorbidities, hospitalization, and transition into ESRD [20]. After dialysis initiation, most of the adverse outcomes occurred within the first year of hemodialysis. The all-cause mortality and mortality due to cardiovascular disease or other causes is found to peaked in the second month after initiation, and then decreased [20]. It is unclear whether the MPE program could extend the financial benefit to after the initiation of dialysis and reduces the medical costs during the first 6 months of hemodialysis initiation. We hypothesize that knowledge acquisition from MPE in the predialysis period may have a “legacy effect” during the postdialysis period. This beneficial effect of MPE may result in differences in disease patterns, reduced medical expenditure and utilization, and reduced medical costs in the immediate postdialysis period. To further clarify this issue, we studied the medical expenditure and utilization incurred during the first 6 months of dialysis initiation in 425 incident hemodialysis patients who were randomized into MPE and non-MPE groups before reaching ESRD.

## Materials and Methods

**Figure 1:**
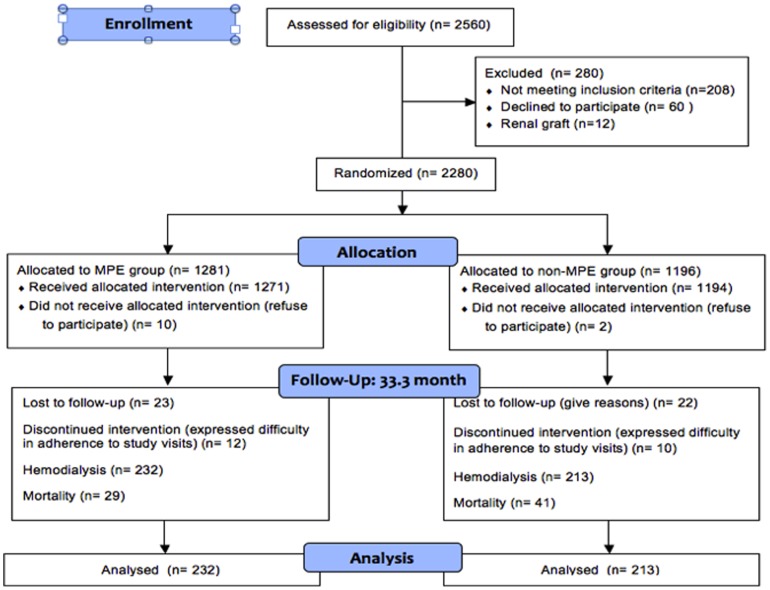
Enrolment scheme and patient status. MPE: multidisciplinary predialysis education.

Patient cohort and settings The protocol for this trial and supporting CONSORT checklist are available as supporting information; see Checklist S1 and Protocol S1. This is an analysis of a subset population from our previously reported randomized cohort (Clinical Trials.gov NCT00644046) [16]. Briefly, the cohort includes predialysis CKD patients with an estimated glomerular filtration rate (eGFR) of <60 mL⋅min^−1^⋅1.73 m^−2^ (determined by using the Modification of Diet in Renal Disease equation) who visited the nephrology outpatient clinics of the Department of Nephrology, Chang Gung Memorial Hospital, Keelung, from July 2007 and followed up to June 30, 2011. Patients aged 18–80 years and without renal graft failure were included in the study after obtaining informed consent from them. A total of 2280 patients were enrolled in the study and were randomly divided into the MPE group and the non-MPE group by using a random table at study entry. Four hundred and five patients reached ESRD needing hemodialysis after a mean follow-up of 33±2.6 months (232 patients in the MPE group and 213 patients in the non-MPE group, as shown in Figure 1). The medical expenditure and utilization in the first 6 months of initiation of hemodialysis in these 425 patients were accurately recorded and compared between MPE and non-MPE patients. Medical service utilization was calculated as the frequency of outpatient visits and the frequency and length of hospitalization. Outpatient visits were categorized as outpatient services, preventive care (e.g., influenza vaccination and dietary counseling), and emergency services. Medical service expenditures included outpatient expenditures (all costs including physicians' and nursing fees, examinations, surgery, and medication) and inpatient expenditures (all costs including laboratory testing, imaging testing, medications, surgery and consulting, ward and administrative, nasogastric tube feeding, and hemodialysis fees). The expenditures for each participant were totaled to compute the sum of ambulatory and inpatient medical service utilization costs and expenditures. The analysis of costs in this study only included those medical costs for which our hospitals made reimbursement claims to the NHI. The salaries, overheads, and administrative costs of the care team were not included. This study was approved by the ethics committee of the institutional review board of Chang Gung Memorial Hospital (Number: 100-0040A3, 96-0408B) and was conducted according to the principles expressed in the Declaration of Helsinki. All patients provided written informed consent. The registration of our cohort at Clinical Trials.gov was delayed by administrative issues (set up of Core Lab, employment of research assistance). The authors confirm that all ongoing and related trials for this drug/intervention were registered.

### MPE

The MPE program was implemented in May 2006 at the Keelung Center. The team comprised a nurse for case management, social workers, dietitians, hemodialysis, peritoneal dialysis patient volunteers and 10 nephrologists. The program consisted of an integrated course involving individual lectures on renal health, delivered by the case-management nurse, according to the guidelines given in a standardized instruction booklet. The lectures focused on nutrition, lifestyle, nephrotoxin avoidance, dietary principles, and pharmacological regimens. Furthermore, the case-management nurse contacted the patients to ensure timely follow-up. Standardized interactive educational sessions were periodically conducted wherein all patients were interviewed depending on their CKD stage, determined earlier by using the NKF/DOQI guideline. Stage III or IV CKD patients were followed up every 3 months, and stage V CKD patients were followed up on a monthly basis. For stage III CKD patients, the program consisted of lectures on healthy renal function, the clinical presentation of uremia, risk factors and complications associated with renal progression, and an introduction to the various renal replacement therapies (i.e., hemodialysis, peritoneal dialysis, and renal transplantation). For stage IV CKD patients, the program included discussions on the management of complications associated with CKD, indications of renal replacement therapy, and the evaluation of vascular or peritoneal access. Patients with stage V CKD were monitored for timely initiation of renal replacement therapies, the care of vascular or peritoneal access, dialysis-associated complications, and registration for inclusion in the renal transplantation waiting list. All patients received dietary counseling biannually from a dietitian. In addition, the case-management nurse often contacted the participants by telephone to encourage them to inform their nephrologists of their symptoms and to reinforce the importance of medical visits. The MPE program was discontinued once renal replacement therapies were initiated for these patients.

### Customary care

The same group of nephrologists instructed all participants about renal function, the evaluation of laboratory data, and clinical indicators of chronic renal failure, as well as about the strategies for its management and treatment. Furthermore, the nephrologists explained the general principles of hemodialysis and peritoneal dialysis when the patients exhibited an eGFR of <30 mL⋅min^−1^⋅1.73 m^−2^ (stage IV CKD). All patients were provided with written instructions. The nephrologists evaluated the comorbidity factors influencing each patient's condition before referral to a nurse specializing in hemodialysis or peritoneal dialysis. The nursing staff provided instructions for daily living and explained the criteria used for hemodialysis and peritoneal dialysis selection, and the difference between the two modalities.

### Statistical analysis

Descriptive statistics were expressed as the mean (standard deviation) or median (interquartile range). Discrete variables were presented in terms of frequencies and percentages. The normality of numerical variables was tested using the Kolmogorov-Smirnov method, and an appropriate transformation was considered before statistical testing. The Student t-test or Mann-Whitney U-test was applied to compare mean or median values among the groups. The association between categorical variables was analyzed by using the χ^2^ test. Multiple linear regression analysis was applied to calculate the unstandardized coefficients associated with MPE in both inpatient and total medical cost. All statistical tests were two-tailed, and a p value <0.05 was considered to be statistically significant. Data were analyzed by using SPSS 17.0 for Windows XP (SPSS Inc., Chicago, IL, USA).

## Results

The mean age of study patients was 63.8±13.2 years, and 221 (49.7%) of them were men. The mean serum creatinine level and eGFR was 6.1±4.0 mg/dL and 7.6±mL⋅min^−1^⋅1.73 m^−2^, respectively, at dialysis initiation. Diabetes mellitus was identified as the leading cause of renal disease in both groups, followed by chronic glomerulonephritis. Table 1 shows the demographic and clinical characteristics of the patients in the MPE and non-MPE groups at the initiation of hemodialysis. These patients received 4 hours of hemodialysis 3 times weekly. Hemodialysis for these patients used single-use hollow-fiber dialyzers equipped with modified cellulose-based, polyamide, or polysulfone membranes. The dialysate used in all patients was a standard ionic composition and bicarbonate-based buffer. Many indices of dialysis adequacy (Urea reduction rate, Kt/V and normalized protein catabolic rate) were similar at baseline for the two groups of patients. However, the MPE group patients were more likely to have permanent vascular access created (61.6% vs. 46.9%, p = 0.002) and less insertion of double-lumen catheter at initiation of hemodialysis (55.6% vs. 45.1%, p = 0.029, Table 1).

**Table 1:** baseline characteristics of study patients.

Parameter	MPE group (n = 232)	Non-MPE group (n = 213)	p
Age, years	67.5±11.4	61.8±15.0	<0.001
Male, n (%)	116 (50.0%)	105 (49.3%)	0.882
Diabetes, n (%)	153 (65.9%)	127 (59.6%)	0.168
Hypertension, n (%)	202 (87.1%)	171 (80.3%)	0.052
Coronary artery disease, n (%)	30 (12.9%)	41 (19.2%)	0.069
Cerebrovascular disease, n (%)	30 (12.9%)	29 (13.6%)	0.832
Gout, n (%)	61 (26.3%)	44 (20.7%)	0.162
eGFR, ml/min	7.49±3.1	7.87±3.6	0.228
Serum albumin, mg/dL	3.1±0.7	3.2±0.8	0.062
Hemoglobin, g/dL	9.4±1.6	9.0±2.7	0.100
Urea reduction rate, %	0.74±0.7	0.76±0.8	0.464
Kt/V, Daugirdas	1.66±0.36	1.71±0.38	0.509
nPCR, g/Kg/day	1.13±0.37	1.20±0.32	0.371
Systolic blood pressure, mmHg	144.3±22.7	147.2±25.9	0.221
Primary renal disease			0.135
Diabetes, n (%)	139 (59.9%)	118 (55.4%)	
Hypertension, n (%)	6 (2.6%)	5 (2.3%)	
Chronic glomerulonephritis, n %	16 (6.9%)	13 (6.1%)	
Others, n %	71 (30.6%)	77 (36.2%)	
Education levels			0.650
Below elementary	42 (18.1%)	3 (1.4%)	
Elementary	151 (65.1%)	207 (97.2%)	
High school	31 (13.4%)	3 (1.4%)	
University	8 (3.4%)	0 (0%)	
Vascular access at initiation of dialysis			
Patients with vascular access created, n (%)	143 (61.6%)	100 (46.9%)	0.002
Patients without insertion of double-lumen catheter, n (%)	129 (55.6%)	96 (45.1%)	0.029

Abbreviation: eGFR, estimated glomerular filtration rate; nPCR, normalized protein catabolic rate.

MPE patients tended to have lower total medical cost in the first 6 months after hemodialysis initiation (9147.6±0.1 USD/patient vs. 11190.6±0.1 USD/patient, p = 0.003, Table 2). Despite the fewer outpatient visits of MPE patients, the cost of outpatient service did not differ between the groups. Most of the costs of outpatient service were spent for treatment (including hemodialysis, laboratory, and image study; Table 3). However, MPE patients were significantly fewer in numbers [mean (SD) 0.61±0.9 vs. 1.0±1.2, p<0.001; median (IQR) 0 (1) vs. 1 (2), p<0.001] and had shorter lengths [mean (SD) 10.6±21.9 days vs. 19.3±29.3 days, p<0.001; median (IQR) 0 (15) vs. 8 (27) days, p<0.001] of hospitalization than non-MPE patients. Therefore, the medical cost of inpatient service was significantly lower in MPE patients [median 0 (2617.4) or (mean 2261.8±5635.8) USD/patient in MPE patients vs. median 1559, 4 (5019.6) or (mean 3698.8±5540.9) USD/patient in non-MPE patients, respectively, p<0.001, Table 2]. The reduced cost of inpatient service observed in MPE patients was attributed to the reduction in the costs of physicians, wards, nasogastric feeding, radiology examination, nursing, blood transfusion, hemodialysis, medication, and pharmacist service fees. Most of the costs of inpatient service were spent on hemodialysis treatment in both groups of patients (Table 4). Eighty-eight (37.9%) patients in the MPE group had at least one hospitalization, compared with 127 patients (59.6%) in the non-MPE group (p<0.001). In all first hospitalizations, 66 (75%) patients in the MPE group were admitted to the nephrology ward. Similarly, 94 (74%) of the non-MPE patients were admitted to the nephrology ward at their first hospitalization. Table 5 lists the main causes of the first hospitalization and surgery in both groups of patients. Cardiovascular disease (including uncontrolled hypertension, coronary artery disease, stroke, heart failure, and peripheral artery occlusive disease) was the main cause of first hospitalization in all patients. Participation in the MPE program reduced cardiovascular hospitalization during the first 6 months postdialysis (18.53% vs. 29.58%, p = 0.007). Among all patients, those in the MPE group were more likely to have fewer vascular access related surgeries during the first admission [35 patients (15.09%) vs. 55 (25.82%), p = 0.005]. Most first surgeries were performed for Hickman catheter–related intervention (implantation, exchange, and removal) and arteriovenous fistula/graft–related intervention (creation, thrombectomy, repair, and excision; Table 5).

**Table 2:** Medical utilization and expenditure between groups.

Parameter	MPE group (n = 232)	Non-MPE group (n = 213)	p
No. outpatient visits, times/patient	15.1±11.6	17.9±11.1	0.009
No. of hospitalization, times/patient	0.00 (1.00)	1.00 (2.00)	<0.001*
Frequency of hospitalization, n (%)			<0.001
Never	144 (62.1%)	86 (40.4%)	
1 time	47 (20.2%)	73 (34.2%)	
2 times	31 (13.4%)	30 (14.1%)	
≥3 times	10 (4.3%)	24 (11.3%)	
Length of hospitalization, days/patient	0.00 (15.00)	8.00 (27.00)	<0.001*
Cost of outpatient service, mean, USD/patient	6885.7±5201.2	7491.8±4200.2	0.175
Log cost of inpatient service, mean, USD/patient	3.09±4.02	4.95±4.15	<0.001
Log total cost of medical service, mean, USD/patient	6.75±4.21	8.58±2.43	<0.001

*p value using Mann-Whitney U test.

**Table 3:** Costs of outpatient service between groups.

Variables	MPE group (n = 232)	Non-MPE group (n = 213)	p
Hemodialysis, laboratory and imagen	6258.8±4647.2	6841.5±3883.1	0.154
Physician fee	94.4±80.1	116.9±93.4	0.007
Medication fee	512.6±120.1	510.8±75.4	0.985
Pharmacist, nursing and administrative fee	19.9±16.4	22.5±17.4	0.107

Costs were expressed in mean ± SD, USD/patient.

**Table 4:** Log-transformed costs of inpatient service between groups.

Variables	MPE group (n = 232)	Non-MPE group (n = 213)	p*
Physician fee	2.03±2.67	3.28±2.79	<0.001
Ward fee	2.49±3.26	3.99±3.38	<0.001
Laboratory examination fee	5.22±1.32	5.52±1.15	0.126
Nasogastric tube feeding fee	0.56±1.61	1.40±2.36	<0.001
Radiology examination fee	1.17±2.05	1.84±2.29	<0.001
Nursing fee	1.78±2.69	2.83±2.91	<0.001
Surgery fee	1.89±3.82	3.31±4.58	<0.001
Blood transfusion fee	1.04±1.93	1.89±2.39	<0.001
Hemodialysis fee	2.65±3.46	4.13±3.58	<0.001
Medication fee	2.16±2.94	3.45±3.08	<0.001
Pharmacist service fee	1.48±1.98	2.32±2.04	<0.001

Costs were expressed in mean ± SD, USD/patient.

**Table 5:** Main cause of first hospitalization and surgery.

	MPE group	Non-MPE group
**Cause of first hospitalization, n**		
Renal-related disease	12 (5.17%)	26 (12.21%)
Cardiovascular disease	43 (18.53%)	63 (29.58%)
Vascular access infection	9 (3.88%)	11 (5.16%)
Acute pulmonary edema	3 (1.29%)	4 (1.88%)
Other	21 (9.05%)	23 (10.8%)
No hospitalization	144 (62.07%)	86 (40.38%)
**Cause of first surgery, n**		
Hickman catheter related	25 (10.78%)	36 (16.9%)
Arteriovenous fistula/graft related	24 (10.34%)	39 (18.31%)
Limb amputation	2 (0.86%)	3 (1.41%)
Hemothorax/pneumothorax	1 (0.43%)	6 (2.82%)
Other	15 (6.47%)	4 (1.88%)
No surgery	165 (71.12%)	125 (58.69%)

Cardiovascular disease includes uncontrolled hypertension, coronary artery disease, stroke, heart failure, and peripheral artery occlusive disease.

Participation in MPE was independently associated with reduced inpatient cost and total medical cost in the first 6 months of dialysis initiation by using various adjustment strategies, including demographic characteristics (age and gender) in model 2, concomitant disease (diabetes mellitus and number of comorbidities) in model 3, baseline biochemistry at entry to hemodialysis (eGFR, hemoglobin, and serum albumin levels) in model 4 and status of use of double-lumen catheter at initiation of hemodialysis (model 5, Table 6).

**Table 6:** Multiple linear regression analysis of effect of MPE on inpatient and total medical cost.

	Inpatient cost		Total cost	
	Unstandardized Coefficients	p	Unstandardized Coefficients	p
Model 1	−43109.74	0.007	−61289.80	0.003
Model 2	−46226.19	0.005	−60200.11	0.005
Model 3	−44575.46	0.005	−62959.92	0.003
Model 4	−44447.13	0.001	−54490.22	0.013
Model 5	−33826.85	0.029	−52241.43	0.011

Both of the two dependent variables in these models were the log transformation of inpatient costs and total costs.

Model 1: crude.

Model 2: adjusted for age and gender.

Model 3: adjusted for diabetes status and number of comorbidities.

Model 4: adjusted for eGFR, hemoglobin and serum albumin levels at initation of hemodialysis.

Model 5: adjusted for insertion of double-lumen catheter at initiation of hemodialysis.

## Discussion

In this prospective study, we examined the medical expenditure and utilization incurred in the first 6 months of dialysis initiation in 425 incident hemodialysis patients who were randomized into MPE and non-MPE groups before reaching ESRD. We found that participation in a multidisciplinary education program at the predialysis period was associated with reduced inpatient service utilization, reduced inpatient cost, and reduced total medical cost in the first 6 months of hemodialysis. The reduction of cost in inpatient service was attributed to the reduction in cardiovascular disease and vascular access–related surgery during hospitalization. The decreased inpatient cost and total medical cost of service associated with MPE were independent of patients' demographic characteristics, concomitant disease, baseline biochemistry at entry to hemodialysis and use of double-lumen catheter at initiation of hemodialysis. The findings of the present study provide evidence supporting the implementation of MPE as part of integrative CKD care to combat the high economic burden and financial impact of hemodialysis on patients.

A previous prospective controlled study demonstrated that MPE based on the NKF/DOQI guidelines effectively reduced the incidence of ESRD and all-cause mortality with a significantly reduced overall hospitalization rate among MPE patients [16]. In our study, the delivery of MPE was stopped once the patient started hemodialysis therapy. After the initiation of hemodialysis, all patients received dietary counseling in the hemodialysis room on a monthly basis. On the basis of these assumptions, knowledge acquisition from a multidisciplinary education program in the predialysis period might be one of the factors that influence the disease pattern and health-care utilization in the postdialysis period. The positive effect of MPE in the postdialysis period could be the result of many factors, such as increased knowledge about self-care [17], better diet and fluid control, medication compliance, better preparation of vascular access, the adoption of a healthier lifestyle, and greater awareness about the use of nephrotoxin. The transition period into ESRD and the first year of dialysis therapy represented the most vulnerable point that demands the highest health-care expenditure and utility in CKD patients [20]. The cost-saving effect of MPE in terms of inpatient and total medical service in this immediate postdialysis period was confirmed by the regression models with the adjustment of different covariates. The total medical cost of the first 6 months of hemodialysis in MPE participants was 18% less than that of nonparticipants (9147.6 USD/patient vs. 11190.6 USD/patient). For a total of 8000 incident hemodialysis patients each year in Taiwan [3], the crude estimation of cost-saving could be up to 16.3 million USD per 6 months if each incident patient was to be provided with the MPE program before reaching ESRD.

MPE is certainly beneficial in lowering the enormous cost for the care of hemodialysis patients. Although the long-term economic impact of MPE has not been studied, the findings of the present study suggest the need for a universal and efficient delivery of multidisciplinary education to all CKD patients as early as possible.

Several studies have reported on the cost-saving effect of MPE in the hospitalization of CKD patients; however, only a few retrospective studies have addressed the financial benefits of MPE in the postdialysis period [18], [19]. From this prospective study, we have found that hemodialysis and the diseases associated with this treatment (cardiovascular disease and noninfectious vascular access complication) were responsible for most of the health-care cost incurred in both inpatient and outpatient services (Tables 3 and 4). Taking into account the beneficial effect of MPE in reducing renal progression [17], the incidence of ESRD [16], and cardiovascular hospitalization and vascular access surgery in the postdialysis period in our study, the implementation of MPE could result in an overall reduction in the medical utilization and expenditure of CKD patients. Similarly, Wei et al., in their retrospective observational study, found that participants in the CKD care program had lower medical costs at dialysis initiation and lower medical cost for the total period of observation than nonparticipants because of the early preparation of vascular access and the lack of hospitalization at dialysis initiation [18]. A retrospective observational study described fewer hospital days and lower total health-care costs during the year after dialysis initiation in patients receiving predialysis nephrology care [19]. We have reported a reduction in the 1-year hospitalization rate (2.8% vs. 16.4%) in recipients of MPE in a controlled prospective cohort. However, the reason for hospitalization for these patients did not differ significantly between them [16]. In a 3-year prospective study, participation in multidisciplinary care was associated with a 40% reduction in the risk of infection-related hospitalization in the predialysis period [21]. A propensity score matched cohort study found that MPE participants had less unplanned urgent dialysis, shorter hospital days, and a lower incidence of cardiovascular events than non-MPE patients during the observation period [22]. A novel finding of our study was that patients with MPE had reduced cardiovascular-related hospitalization in their postdialysis period. This finding could possibly be attributed to better control of cardiovascular risk factors, better medical adherence, fluid control, and compliance with dietary restriction in the postdialysis period with a prior acquisition of renal knowledge.

Our study included a comprehensive analysis of medical service utilization and expenditures with detailed information about outpatient and inpatient costs in patients with comparable baseline characteristics derived from a randomized cohort. The delivery of MPE was standardized according to the NKF/DOQI guidelines. A single nurse conducted the MPE program for all patients to limit interpersonal variability. This single-center study has been conducted in a university-afflicted teaching hospital. All enrolled participants were patients who visited the nephrology outpatient clinics of Chang Gung Memorial Hospital at Keelung and underwent dialysis treatment at the hemodialysis unit of the same hospital. This hospital is a group practice institution comprised 10 nephrologists and only one hemodialysis unit. Furthermore, variation within dialysis units did not exist, because the single hemodialysis unit setting. In this group practice setting, all nephrologists followed the same institutional regulation. All nephrologists were subject to the same criteria of reimbursement under a single NHI system of government of Taiwan. These 10 nephrologists took care of all renal patients at outpatient and inpatient departments. The same group of 10 nephrologists instructed the MPE and non-MPE patients at their predialysis education and also continuous their medical care in the post-dialysis period at our hemodialysis unit. For these reasons, confounding effects by practice pattern of individual nephrologists might be neglected. Despite the several advantages of MPE presented in our investigation, several limitations should be addressed. First, the study represented a secondary cost analysis of a subset of patients who started hemodialysis and were randomized into MPE and non-MPE groups before reaching ESRD. Second, patients who had died before reaching ESRD were excluded. It was unclear how MPE could influence the postdialysis health-care expenditure and utility costs of these critically ill patients. Third, the age of patients in the two groups was not comparable at the baseline. However, despite the older age of the MPE patients, the outcome of interest was consistently better than that of the non-MPE patients. In addition, the adjustment for age in the regression model has indicated no significance of this factor in our outcome. Finally, although this study examined the economic impact of MPE for the first 6 months of dialysis, its long-term effect remains unclear. Further investigations with a large-scale population, including other treatment modalities (peritoneal dialysis or renal transplantation) and a longer duration would be needed to demonstrate the cost benefit of MPE for an extended period.

In conclusion, in this prospective study, we demonstrated the lowered inpatient and total medical costs in the first 6 months postdialysis in patients receiving the MPE program. This cost reduction was attributed to decreased inpatient service utilization, and principally concerning services used because of cardiovascular causes and vascular access–related surgeries. This reduction in cost was independent of the patients' demographic characteristics, concomitant disease, baseline biochemistry and status of use of double-lumen catheter at entry to hemodialysis. This valuable information confirmed the legacy effect of the MPE program on the economic outcome in the postdialysis period. Although the optimal dose and duration of MPE remains debated, an efficient and universal delivery of multidisciplinary education should be considered as part of the integrative care of CKD patients. This simple strategy could be an ideal resolution to the problem of the increasing financial burden of renal failure worldwide.

## Supporting Information

Checklist S1 **Consort checklist.**
(DOC)

Diagram S1 **CONSORT 2010 Flow Diagram.**
(DOC)

Protocol S1 **Trial protocol.**
(DOC)
