# Supplementary material for: Multidisciplinary Predialysis Education Reduced the Inpatient and Total Medical Costs of the First 6 Months of Dialysis in Incident Hemodialysis Patients
Source: PLoS One. 2014 Nov 14;9(11):e112820. doi: 10.1371/journal.pone.0112820 (PMC4232513; doi:10.1371/journal.pone.0112820)
Supplement: Diagram S1 — CONSORT 2010 Flow Diagram. (DOC) [file pone.0112820.s002.doc]

**
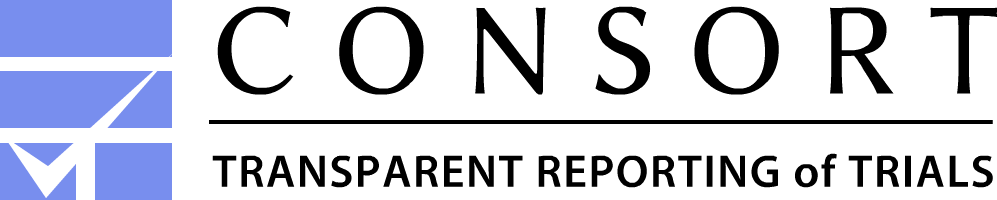
**

**Allocation**

**Analysis**

**Follow-Up: 33.3 month**

**Enrollment**

Assessed for eligibility (n= 2560)

Excluded (n= 280)

♦  Not meeting inclusion criteria (n=208)

♦  Declined to participate (n= 60 )

♦  Renal graft (n=12)

Analysed (n= 232)

Lost to follow-up (n= 23)

Discontinued intervention (expressed difficulty in adherence to study visits) (n= 12)

Hemodialysis (n= 232)

Mortality (n= 29)

Allocated to MPE group (n= 1281)

♦ Received allocated intervention (n= 1271)

♦ Did not receive allocated intervention (refuse to participate) (n= 10)

Lost to follow-up (give reasons) (n= 22)

Discontinued intervention (expressed difficulty in adherence to study visits) (n= 10)

Hemodialysis (n= 213)

Mortality (n= 41)

Allocated to non-MPE group (n= 1196)

♦ Received allocated intervention (n= 1194)

♦ Did not receive allocated intervention (refuse to participate) (n= 2)

Analysed (n= 213)

Randomized (n= 2280)

**CONSORT 2010 Flow Diagram**
