# Supplementary material for: Multidisciplinary Predialysis Education Reduced the Inpatient and Total Medical Costs of the First 6 Months of Dialysis in Incident Hemodialysis Patients
Source: PLoS One. 2014 Nov 14;9(11):e112820. doi: 10.1371/journal.pone.0112820 (PMC4232513; doi:10.1371/journal.pone.0112820)
Supplement: Protocol S1 — Trial protocol. (DOC) [file pone.0112820.s003.doc]

| **Study protocol (CMRP study protocol)**  **Study Title:** Chronic Kidney Disease Prevention of an-Lo District, Keelung.  **Principal Investigate:** I-Wen Wu, M.D.  **Study Outline and Abstract:**  The prevalence and the incidence of the end-stage renal disease (ESRD) are extremely high in Taiwan (1, 2). More than 45,000 patients are under renal replacement therapy in the year 2004 (2). The disease had not only caused a significant impact in personal life, but also a great burden on social security and government-run health insurance. However, despite this high prevalence, the awareness of chronic kidney disease (CKD) in general population remains low (3, 4). The patients always come out too late for the intervention to slow down the progression of renal failure. Furthermore, most of them are not well prepared for the renal replacement therapy. The facts result in high mortality and morbidity in this specific population (5). It is mandatory to screen out and treat these patients early enough. However, these patients are deep into the community as the asymptomatic nature of CKD.  The major purpose of the study is to screen the community for the early CKD and provide the appropriate intervention at time. The study will collect the characteristic demographic epidemilogical data and find out risk factors for CKD of this geographic area, provide multidisciplinary education of CKD and establish timely referral for appropriate nephrologist care for treatment and monitoring of complications.  The project will be last for 3 years. We will start to screen the patients within the community to identify the CKD population. This population will randomized into two group (one group with standard nephrologist care and another group with standard nephrologist care plus multidisciplinary CKD education) to evaluate the effectiveness of CKD education on the progression of renal function. The primary end-point will be the doubling tine estimated GFR, development of end-stage renal disease and all cause mortality. The non-CKD group of community will also follow up for urine and blood test for renal function, to determine the incidence and risk factors of new-onset renal disease. The accomplishment of this study will retard renal progression, improve community health and establish a model in preventing the chronic kidney disease. The included patients will be followed in 5 aspects as described in the following section. The secondary targets will be described in each sub-project.  This sub-project will focus on the basic screening, staging, treatment, education and follow-up of these CKD patients. We will find out the sub-clinical patients in the community. Standard therapy will be given by the aid of a specific case-control nurse and nephrologists. All the basic data will be collected and analyzed in these patients. We will finally analyze the effect of active intervention in the progression of CKD.  The project will provide a model of multi-disciplines involvement of the prevention of chronic kidney disease deep into the community.  **Keywords：** Chronic Kidney Disease, prevention, cardiovascular disease References  1. Atkins RC. The changing patterns of chronic kidney disease: the need to develop strategies for prevention relevant to different regions and countries. Kidney International - Supplement. (98): S83-5, 2005 2. Atkins RC. The epidemiology of chronic kidney disease. Kidney International - Supplement. (94): S14-8, 2005 3. Hsu CC, Huang SJ, Wen CP, et al. High prevalence and low awareness of CKD in Taiwan: a study on the relationship between serum creatinine and awareness from a nationally representative survey. Am J Kidney Dis. 48(5): 727-38, 2006 4. Kuo HW, Tsai SS, Tian MM et al. Epidemiological feature of CKD in Taiwan. Am J Kidney Dis. 49: 46-55, 2006 5. Sarnak MJ, Levey AS. Cardiovascular disease and chronic renal disease: A new paradigm. *Am J Kidney Dis* 2000; 35:S117-S131. |
| --- |

| **Background:**  The high incidence and prevalence of End Stage Renal Disease (ESRD) in Taiwan cause impairment of quality of life in dialysis patient and significant socio-economic impact. The Chronic Kidney Disease (CKD) is a detectable and treatable disease that deserves especial medical and socio-epidemiological attention to prevent the progression to ESRD. In fact, most medical and economic efforts are dedicated in the secondary prevention of ESRD but early detection and primary prevention of CKD are rarely emphasized.  **Objectives:**  This study is emphasized in the basic prevention, including screening, evaluation, intervention, follow-up and education of the CKD. The objectives of the study are:   1. Establish epidemiological data of CKD of this District and compare between other region and countries, to find out the possible risk factors of high incidence of CKD in Taiwan. 2. Provide adequate preventive education to retard progression of CKD. 3. Establish and provide facilities for early referral and treatment of CKD. 4. Evaluate and monitor the complication and progression of CKD. |
| --- |

| **Materials and Methods:**  **A) Study population:**  Both the asymptomatic CKD and non-CKD patients identified from the community are included in this study. Periodical preventive and educational programs of CKD will be developed in the community to alert the interest and incentives of citizen about the importance of a health-kidney life. Screening tests including questionnaires, anthropometric measure, evaluation of blood pressure and laboratory tests. The patient will be stratified in to the five stages of CKD according to the estimated glomerular filtration rate. The CKD group will randomized into two group (one group with standard nephrologist care and another group with standard nephrologist care plus multidisciplinary CKD education) to evaluate the effectiveness of CKD education on the progression of renal function. The primary end-point will be the doubling tine estimated GFR, development of end-stage renal disease and all cause mortality. The non-CKD group of community will also randomize, match for age and gender, and follow up for urine and blood test for renal function, to determine the incidence and risk factors of new-onset renal disease. (Table 1)  **B) Evaluation:**  The questionnaires include the personal data (name, address, telephone number, age, gender, marital status, occupation and education level), past history, family history, frequent complaints, dietary and nutritional diary, type and frequency of exercise, medication history and social-psychological concerns of the patient. Anthropometric measure evaluates the body weight and the height. Both the systolic and the diastolic blood pressure are recorded.  **C) Laboratory tests:**  The blood and urine of patient are collected for evaluation. The samples are sent for:  Blood test: Hb/Hct, Fasting Sugar, HbA1c, Bun, Cr, Na, K, Cl, Ca, P, CO_2_, iPTH, renin, aldosterone, albumin, hs-CRP  Urinary test: microalbuminemia or proteinuria  **D) Staging:**  All demographic laboratory data are collected. The patients are classified into the five stages of CKD based on the estimated glomerular filtration rate. Regular follow-up and intervention are developed according to the stage of CKD.  **E) Prevention of CKD:**  **1.** Standard nephrologist care:  I. Non-pharmacological intervention: treat obesity, restrict alcohol, smoking and salt intake, encourage physical activity and exercise, avoid drugs abuse  II.Pharmacological intervention: blood pressure control (< 130/80 mmHg), ACEI/ARB use, lipid profile control, glycemic control (Glycohemoglobin < 6.0), use of paricalcitol.  2. Multidisciplinary CKD education: teaching of nephrotoxins, dietary control, recall for importance of periodic follow-up, by case-control nurse in one by one fashion.  **F) Statistical Analysis:**  Descriptive statistics were expressed as means and standard deviation. Discrete variables were represented as frequencies and group percentage. All variables were tested for normal distribution using the Kolmogorov-Simirnov test. The Student’s *t*-test was applied to compare means of continues variables and normal distribution data. Categorical data were tested using the Chi-square test. Analysis of variance (ANOVA) test with the Turkey *post hoc* test for numerical values, and the Chi-square test for trends were applied to assess categorical data associated with CKD classification. All statistical tests were two-tailed, and a significance level of *p<*0.05 was used.  **References**   - 1. Atkins RC. The changing patterns of chronic kidney disease: the need to develop strategies for prevention relevant to different regions and countries. Kidney International - Supplement. (98): S83-5, 2005   2. Atkins RC. The epidemiology of chronic kidney disease. Kidney International - Supplement. (94): S14-8, 2005   3. Bello AK. Nwankwo E. El Nahas AM. Prevention of chronic kidney disease: a global challenge. Kidney International - Supplement. (98): S11-7, 2005   4. De Franscisco ALM, Fresnedo DF, Palomar R, et al. The renal benefits of a health lifestyle. Kidney international-supplement (99): s2-6, 2005   5. Go AS, Chertow G. M., Fan D, et al. Chronic Kidney Disease and the Risk of Death, Cardiovascular Events and Hospitalization. NEJM, 351:1296-1305, 2004   6. Levin A. The need for optimal and coordinated management of CKD. Kidney international-supplement (99): s7-10, 2005   7. O’Connell, B. Eating right for healthy kidneys. Diabetes Self Manag. 19(1): 39-45, 2002   8. Wang H. Zhang L. Lv J. Prevention of the progression of chronic kidney disease: practice in China. Kidney **In**ternational - Supplement. (94): S63-7, 2005   9. Zandi-Nejad K. Brenner BM. Primary and secondary prevention of chronic kidney disease. Journal of Hypertension. 23(10): 1771-6, 2005   10. 台灣腎臟醫學會腎臟保健推廣機構衛教作業規範 |
| --- |

**Table 1: Study flow chart.**

**
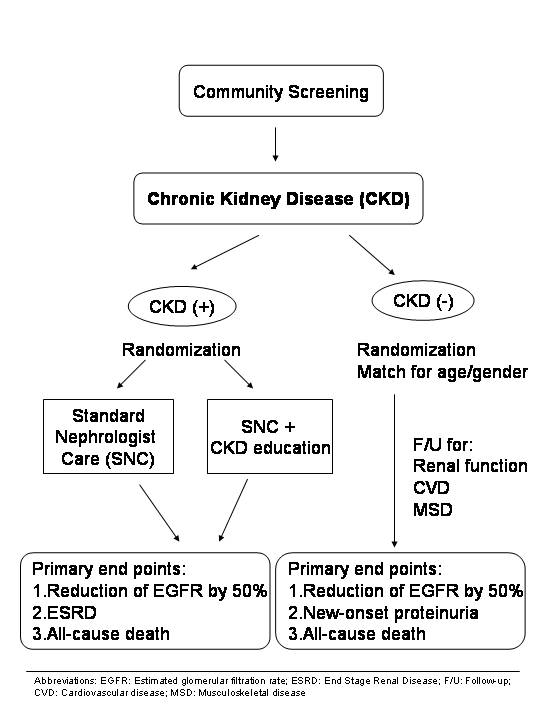
**
